# Exploring knowledge and attitudes toward non-communicable diseases among village health teams in Eastern Uganda: a cross-sectional study

**DOI:** 10.1186/s12889-017-4954-8

**Published:** 2017-12-12

**Authors:** Temitope Tabitha Ojo, Nicola L. Hawley, Mayur M. Desai, Ann R. Akiteng, David Guwatudde, Jeremy I. Schwartz

**Affiliations:** 1 Department of Chronic Disease Epidemiology, Yale School of Public Health, 60 College Street, P.O. Box 208034, New Haven, CT 06520-8034 USA; 2 Uganda Initiative for Integrated Management of Non-Communicable Diseases, Upper Mulago Hill, Kampala, Uganda; 3 Department of Epidemiology and Biostatistics, Makerere University School of Public Health, Kampala, Uganda; 4 Section of General Internal Medicine, Yale School of Medicine, 333 Cedar Street, New Haven, CT 06510 USA

**Keywords:** community health workers, Village health teams, Non-communicable diseases, Uganda, Task-shifting, Community engagement, Health systems

## Abstract

**Background:**

Community health workers are essential personnel in resource-limited settings. In Uganda, they are organized into Village Health Teams (VHTs) and are focused on infectious diseases and maternal-child health; however, their skills could potentially be utilized in national efforts to reduce the growing burden of non-communicable diseases (NCDs). We sought to assess the knowledge of, and attitudes toward NCDs and NCD care among VHTs in Uganda as a step toward identifying their potential role in community NCD prevention and management.

**Methods:**

We administered a knowledge, attitudes and practices questionnaire to 68 VHT members from Iganga and Mayuge districts in Eastern Uganda. In addition, we conducted four focus group discussions with 33 VHT members. Discussions focused on NCD knowledge and facilitators of and barriers to incorporating NCD prevention and care into their role. A thematic qualitative analysis was conducted to identify salient themes in the data.

**Results:**

VHT members possessed some knowledge and awareness of NCDs but identified a lack of knowledge about NCDs in the communities they served. They were enthusiastic about incorporating NCD care into their role and thought that they could serve as effective conduits of knowledge about NCDs to their communities if empowered through NCD education, the availability of proper reporting and referral tools, and visible collaborations with medical personnel. The lack of financial remuneration for their role did not emerge as a major barrier to providing NCD services.

**Conclusions:**

Ugandan VHTs saw themselves as having the potential to play an important role in improving community awareness of NCDs as well as monitoring and referral of community members for NCD-related health issues. In order to accomplish this, they anticipated requiring context-specific and culturally adapted training as well as strong partnerships with facility-based medical personnel. A lack of financial incentivization was not identified to be a major barrier to such role expansion. Developing a role for VHTs in NCD prevention and management should be a key consideration as local and national NCD initiatives are developed.

## Background

The rising prevalence of non-communicable diseases (NCDs) and associated mortality in low- and middle-income countries (LMICs) is well established [1, 2]. Given the limited health and economic resources in these settings, effective, scalable strategies for addressing NCDs are urgently needed [3–6].

Uganda is an example of an LMIC experiencing a growing burden of NCDs. The first nationally representative study of NCDs and their associated risk factors, completed in 2014 using the WHO STEPwise approach (STEPS), revealed that 25.8% of Ugandan men and 22.9% of women had hypertension; 9.5% of men and 19.5% of women were overweight (BMI ≥ 25 kg/m^2^); 4.6% of participants were obese (BMI ≥ 30 kg/m^2^); 3.3% had raised fasting glucose including diabetes; 6.7% had raised total cholesterol levels and 11% were current smokers [7–9]. In addition to cardiovascular disease, diabetes, chronic lung disease, and cancer, the Uganda Ministry of Health (MOH) considers sickle cell disease, injury/disability, gender-based violence, mental health, substance use, oral health, and palliative care to be other NCD priority areas [10]. However, a nationwide needs assessment of health facilities’ readiness to deliver NCD care, revealed large gaps in human resource readiness to treat NCDs [11].

In Uganda and other LMICs, Community Health Workers (CHWs) are increasingly being mobilized to address chronic communicable diseases [12, 13] and NCDs through task-shifting to fill the human resource gap for these health services [14, 15]. CHWs in Uganda are organized into Village Health Teams (VHTs), which comprise the first tier of the referral hierachy in the public health sector. VHTs are volunteers recommended by their communities [16] and with basic health training lasting 5-7 days [16, 17], they serve as the initial point of contact for healthcare services in their communities. While VHTs are heavily involved in community mobilization, disease prevention, and health promotion for communicable diseases and maternal and child health [16], there is currently no NCD component to their role.

We sought to determine whether incorporating NCD-related activities into the VHT role may be possible in Uganda by assessing the knowledge, perceptions, and attitudes of VHT members toward NCDs. Specifically, we aimed to identify perceived facilitators of and barriers to NCD-related work and to identify potential roles for VHTs in NCD prevention and care in Uganda.

## Methods

### Study design and location

We conducted a cross-sectional mixed methods study of VHT members within the Iganga-Mayuge Health Demographic Surveillance Site (IMHDSS) in Eastern Uganda (June-August 2015). IMHDSS, a designated site for community-based research founded by Makerere University, has a population of approximately 80,000 people, across 65 villages within Iganga and Mayuge districts [18]. The IMHDSS is largely rural, but includes peri-urban areas around Iganga and Mayuge town centers.

### Data collection

We conducted a questionnaire and focus group discussions (FGDs) with VHT members witihin IMHDSS. Six field assistants, fluent in English and Lusoga (the local language), administered questionnaires, facilitated FGDs, and obtained written informed consent from participants. The field assistants, who had conducted field work in IMHDSS for an average of 6 years, recieved 2 weeks training for this study. All study tools were translated into Lusoga and back-translated into English to ensure no loss of meaning during translation. The questionnaire and consent forms were pre-tested with 6 VHT members and revised based on feedback received.

### Questionnaire

During the study period, 81 eligible VHT members were working in IMHDSS; we randomly selected 68 to answer the questionnaire. The 68 randomly selected were representative of the larger group on terms of gender and village of operation. The interviewer-administered questionnaire included socio-demographic characteristics and 30 NCD-related knowledge, attitudes, and practices (KAP) questions. The KAP questions were drawn from the 2014 Uganda STEPS survey, and from a validated instrument previously used in Mongolia [9, 19]. Responses to knowledge questions were generally yes/no/don’t know or Likert scales (see Table 3 for examples). Where knowledge was tested, “don’t know” and missing responses were included with incorrect answers.

Given that there was no published literature that stated the proportion of VHTs expected to have existing knowledge of NCDs and their risk factors, the sample size was determined by assuming that 50% of VHTs (with ± 0.05 error) would have existing knowledge of NCDs, at a significance level of 0.05.

### Focus group discussions

We invited 36 VHTs to participate in FGDs; 33 (92%) agreed to participate and were divided among four FGDs. Of the 33 FGD participants, 26 (79%) also completed the KAP questionnaire. FGD participants were purposively selected based on their sex and the district in which they operated. According to a 2015 national VHT assessment in Uganda, communities respond differently to male and female VHT members [16]; thus, we conducted same-sex FGDs. Each of the groups included six to eleven participants. One FGD with each sex was held in each district. Field assistants utilized a nine-question interview guide to lead FGDs (see example questions in Table 4). Information collected through the FGDs sought to complement the questionnaire data by eliciting VHT members’ perceptions of NCDs as an important health issue and their potential roles in tackling the problem of NCDs in their communities. Each FGD lasted 45-60 minutes and was audio-recorded.

The study protocol, data collection tools, and consent forms were reviewed and approved by the Yale University Human Subjects Committee, the Higher Degrees Research and Ethics Committee at Makerere University School of Public Health, and the Uganda National Council of Science and Technology.

### Data management and analysis

#### Questionnaire

Questionnaire data were double-entered and checked for consistency. R software (version 3.1.2, R Foundation for Statistical Computing, 2014) was used to calculate descriptive statistics for study variables.

#### Focus group discussions

FGDs were transcribed and translated from Lusoga to English by the field assistants. Transcripts were checked against the audio recordings and cleaned to remove identifying information. After an initial reading of the transcripts and field notes, codes were developed based on the main interview questions, prior literature, and emergent concepts from the data. Two investigators (TTO and NLH) independently reviewed one transcript and developed a coding structure, which was discussed and clarified, before an initial coding scheme was agreed upon. The coding scheme was refined iteratively as each transcript was reviewed; changes to the original coding scheme were applied to all transcripts. Each transcript was coded independently by both investigators, who met to reach consensus.

We conducted a thematic analysis where individual codes were read in aggregate and a written summary created. We identified nine main and 44 sub-codes, which were merged into four themes: 1) VHTs understanding of NCDs; 2) VHTs role in preventing/treating community NCDs; 3) facilitators of their role; and 4) barriers to their role. The analysis attempted to achieve equal and fair representation of the participant’s opinions. We selected representative quotes to illustrate study findings and retained colloquial language.

## Results

### Questionnaire

As shown in Table 1, participants ranged from 28 to 66 (mean 43.6) years of age. Approximately two-thirds were female and received secondary school education. Participants had worked as VHTs for an average of 6.4 years and spent 19 hours per week doing VHT work.

**Table 1:** Sample characteristics of Village Health Team (VHT) members who completed knowledge, attitudes, and practice questionnaires and focus group discussions.

Variable	Mean ± SD (range) or N (%)				
	KAP participants (*N*=68)	FGD 1 (*N*=11)	FGD 2 (*N*=8)	FGD 3 (*N*=8)	FGD 4 (*N*=6)
Age	43.6 ± 7.7 (28-66)	42.6±6.8 (32-53)	44.4±9.4 (31-60)	45.5±5.1 (38-54)	41.3±8.0 (35-56)
Sex					
Male	19 (27.9)	0 (0.0)	8 (100.0)	0 (0.0)	6 (100.0)
Female	49 (72.1)	11 (100.0)	0 (0.0)	8 (100.0)	0 (0.0)
Highest education level					
Primary	13 (19.1)	1 (9.1)	0 (0.0)	1 (12.5)	3 (50.0)
Secondary	51 (75.0)	10 (90.9)	8 (100.0)	6 (75.0)	1 (16.7)
Post-secondary	4 (5.9)	0 (0.0)	0 (0.0)	1 (12.5)	2 (33.3)
Occupation					
Farming	30 (44.1)	7 (63.6)	8 (100.0)	3 (37.5)	2 (33.3)
Business	19 (27.9)	3 (27.3)	0 (0.0)	2 (25.0)	3 (37.5)
Housewife	6 (8.8)	1 (9.1)	0 (0.0)	2 (25.0)	0 (0.0)
Other	13 (19.2)	0 (0.0)	0 (0.0)	1 (12.5)	1 (12.5)
Average VHT work hours (per week)	19.0 ± 11.6	13.9±5.1	15.5±5.6	17.5±9.8	11.2±3.0
Average no. of years as a VHT member	6.4 ± 4.1	6.5±1.5	6.7±1.8	5.2±4.0	8.8±4.6

Nearly all participants (94.1%) knew that NCDs are not transmissible, and 82.4% agreed/strongly agreed that NCDs are common in Uganda (Table 2). The majority of participants claimed to know ‘a little’ about high blood pressure (70.6%), heart disease (61.8%), stroke (52.9%), and type II diabetes mellitus (63.2%), and nearly 90% thought that CVD is becoming more common in Uganda. In addition, 77.9% responded that diabetes is caused by high blood sugar levels, and over half reported that diabetes can cause complications. Thirty-two participants (47.1%) thought diabetes was preventable.

**Table 2:** Frequency distribution of VHT responses to key KAP questions

Question	Question type	Responses N (%)
*General Knowledge and Attitudes related to NCDs*		
A non-communicable disease is one that cannot be spread between people	Knowledge	Yes- 64 (94.1)
Non-communicable diseases are common amongst Ugandans	Attitude	In agreement 56 (82.4) In disagreement 12 (17.6)
*Nuanced Knowledge about specific NCDs*		
*High Blood Pressure- HBP*		
How much do you know about high blood pressure?	Knowledge	Nothing or only heard the term before 10 (14.7) A little about it 48 (70.6) Familiar with it 10 (14.7)
*Cardiovascular disease- CVD*		
How much do you know about heart diseases?	Knowledge	Nothing or only heard the term before 23 (33.8) A little about it 42 (61.8) Familiar with it 3 (4.4)
How much do you know about stroke?	Knowledge	Nothing or only heard the term before 27 (39.7) A little about it 36 (52.9) Familiar with it 5 (7.4)
In general, do you think cardiovascular diseases are becoming more or less common in Uganda?	Knowledge (perceived)	More common 61 (89.7) Less common 4 (5.9) Don’t know 3 (4.4)
*Diabetes- DM*		
How much do you know about diabetes?	Knowledge	Nothing or only heard the term before 16 (23.6) A little about it 43 (63.2) Familiar with it 9 (13.2)
Diabetes is when there is too much sugar in the blood Diabetes complications include: Loss of sensation to the feet Damage to the heart Blindness Diabetes can be prevented	Knowledge	In agreement 53 (77.9) In agreement 34 (50.0) In agreement 43 (64.2) In agreement 46 (67.6) In agreement 32 (47.1)
*Knowledge, Attitudes and Practices on NCD Behavioral Risk Factors*		
*Smoking*		
Does active smoking affect your health?	Knowledge	Yes 68 (100)
Does smoking harm your lungs?	Knowledge	Yes 68 (100)
Does smoking harm your heart?	Knowledge	Yes 55 (80.9)
Do you think smoking around others could harm their health?	Knowledge (Perceived)	Yes 66 (97.1)
As a VHT member, do you talk to community members about the harms of smoking?	Practice	Sometimes or Always 56 (82.4) Never 12 (17.6)
*Alcohol Use*		
In general, when Ugandans drink alcohol, they tend to drink large amounts at once.	Attitude	In agreement 60 (88.2) In disagreement 8 (11.8)
On which occasions would Ugandans drink large amounts of alcohol?	Knowledge (perceived)	At celebration 45 (66.2) After receiving income 43 (63.2) No special reason 40 (58.8) With friends/family 37 (54.4) As a customary act 23 (33.8)
Have you ever advised community members about the harms of drinking alcohol?	Practice	Yes 57 (83.8) No 11 (16.2)

All participants thought smoking affected one’s health and was harmful to the lungs. Approximately 80% thought smoking was harmful to the heart and reported talking to community members about the harms of smoking. Similar numbers of participants reported having advised community members about the harms of excessive alcohol use.

### Focus group discussions

The socio-demographic characteristics of FGD participants was similar to those who completed the questionnaire (Table 1).

Participants’ responses to FGD questions were categorized into four main themes, which are described below; illustrative questions related to each theme are in Table 3.

**Table 3:** Focus Group Discussions (FGDs): themes and corresponding questions

1. VHT Understanding of NCDs What is your understanding of what a non-communicable disease is? [Probe: Could you give me some examples of NCDs?] How often do you see people in your communities with NCDs while doing your VHT work? Are people in your communities aware of NCDs or know of what causes them? [Probe: Why do you think people are not aware of NCDs or their causes?]
2. VHT Role in Preventing NCDs in the Communities As a VHT, do you think NCDs are important health issues that should be taken care of in your communities? What ways could you help in dealing with NCDs in your communities? [Probe: Could you give examples of ways NCDs could be prevented in your communities? How could you manage to help deal with NCDs with your VHT work?]
3. Facilitators of VHT Role in Preventing NCDs in the Communities What would encourage NCDs prevention in your communities? [Probes: What could your communities do? What could VHT workers do to prevent NCDs?]
4. Barriers to VHT Role in Preventing NCD in the Communities What might be the barriers to dealing with NCDs in your communities? [Probe: As VHT workers, what are the barriers to dealing with NCDs in your communities?] [Examples of response-specific probes: e.g. respondent mentions equipment – what kind of equipment do you need that you don’t have? Respondent mentions lack of engagement with health facilities – what are the specific problems that you face?]

#### VHT understanding of NCDs

Asked about their understanding of NCDs, participants either gave examples of specific diseases or spoke to mode of transmission. Diabetes, high blood pressure, heart disease, and cancer were frequently mentioned as examples of NCDs. Some less frequently suggested were ulcers, anemia, and asthma. Participants were confident that NCDs are not transmitted between individuals through environmental or physical contact.

*“These are diseases that cannot be transmitted from one person to another. For example, if you share the same taxi with someone with NCD, it can’t be transmitted.”* FGD2, Participant 1

As discussions progressed, it became evident that many FGD participants had more nuanced knowledge of these diseases; for example, some participants spoke to lifestyle risk factors associated with NCDs, particularly poor diet, or susceptibility based on family history.

*“Some people are not even aware that such diseases exist. You could meet someone who puts a lot of sugar in a small cup of tea, like adding 8 teaspoons in a very small cup of tea. Yet, this person would be at a high risk of getting diabetes.”* FGD1, Participant 7

Importantly, they were aware that NCDs were chronic conditions, often symptomatic only after a long period of latency, which they identified as a possible contributor to the lack of awareness about NCDs in their communities.

*“Since NCDs are diagnosed after a very long time, they tend to affect more because it is only when one sees the signs that one goes to the hospital and it would be too late to prevent.”* FGD4, Participant 3

Participants reported seeing people with NCDs while carrying out their VHT work. However, they reported little or no community awareness of NCDs and no knowledge of the causes, signs, and symptoms. Participants attributed this lack of awareness to a lack of knowledge and a lack of education about NCDs being directed at community members.

#### VHT role in preventing/treating NCDs in communities

Participants unanimously agreed that NCDs are very important health issues needing to be urgently addressed. They regarded themselves as health “connectors” who linked their communities to health services and care and as conduits of knowledge to their communities, provided that they receive training on NCD issues.

*“As VHT, if we can get enough knowledge on NCDs, we can return to the communities and teach our people about NCDs.”* FGD1, Participant 5

Even without any formal training on NCDs, some participants expressed that they already take actions to address NCDs by counseling community members to go for regular check-ups or referring them to health centers.

*“When doing my VHT work, if I find someone with an NCD, I refer them to the hospital for tests.”* FGD1, Participant 6

However, the importance of a working partnership and positive relationship with medical personnel was consistently raised. Participants expressed the need for medical personnel to initate conversations about NCDs by coming to the communities. In doing so, they would foster a safe space to address NCD needs rather than having community members travel to health centers. Thereafter, they felt they could work as the ‘go-betweens’, facilitating continued conversation and transferring information between their communities and medical personnel.

*“If medical personnel could organize workshops in communities to inform them about NCD, it will help in dealing with NCD.”* FGD4, Participant 3

#### Facilitators to a VHT role in preventing/treating NCDs in communities

Participants identified NCD education as the foremost tool they need to possess in order to address NCDs in their communities. Other structural changes they recommended were the availability of screening services and endorsement and collaboration of medical personnel with VHTs. As described above, they emphasized the need for medical personnel to take up active roles through community outreach activities. Importantly, they also felt that visible partnerships between medical personnel and themselves would boost VHT’s credibility in the community and promote their work as VHTs.

*“If medical personnel can come to our villages and inform us, it will ease our work as VHT. At least, they (community) will know that it was the personnel who have taught the VHT about NCD.”* FGD3, Participant 3

According to participants, VHT’s role in NCD prevention and care would be facilitated primarily through education, screening services, proper referral and reporting tools, and medical personnel involvement. Participants noted that uniforms would help validate their positions in the eyes of their communities. Monetary support was deemed less essential. Table 4 provides further illustrative quotes highlighting such facilitators.

**Table 4:** Examplary statements on classification of facilitators to VHT role in preventing NCDs in communities

Structural Facilitators	Exemplary Statements
VHT-NCDs education/VHT knowledge sharing	Ppt#2- FGD1 “At least as VHTs, we should get trained about NCDs as it will help us communicate to the communities; hence NCDs can be prevented within the communities.”
Availability of screening services	Ppt#1- FGD 2 “The nearest health centers to the villages should have equipment to test for NCDs.”
Medical personnel endorsement and collaboration with VHT	Ppt#6- FGD 3 “At least, once in a while, if medical workers could come to the villages, ask us as VHTs to mobilize the community for testing, it will help in prevention.”
Functional Facilitators	
Proper referral and record-keeping tools for VHT	Ppt#5- FGD 2 “At VHTs, if we can keep records of what we have been doing in the prevention of NCDs, and how much coverage one has accomplished, we would be able to reach the whole community, leaving no one out. If we get people with NCDs in the communities, such people can be referred to health centers.”
Availability of medication and education for referred community members	Ppt#5- FGD 4 “Having enough medications at the health centers will help prevent NCDs in our communities.”
VHT transportation and monetary aid	Ppt#6- FGD 3 “I would like monthly payments as they would help me in carrying out preventive work for NCDs. I also need a means of transport (bicycles) and bag to carry my records/register. Since my village is quite big, a bicycle will make it easier for me to move around.”
Friendly attitude from medical personnel	Ppt#10- FGD 1 “If at least, if medical workers are friendly people at the health units such as when people go for treatment and they are treated well, it could help in the prevention of such diseases.”
Training of respected community leaders	Ppt#3- FGD 1 “Employers of VHTs should involve religious leaders in their trainings. In most cases, people listen and honor their religious leaders, local councilors.”
VHT uniforms/increased respect	Ppt#2- FGD 3 “We need uniforms like T-shirts for easier recognition in the communities; this will help in the prevention of NCDs.”
VHT carrying medication/equipment	Ppt#3- FGD 4 “Provide VHT with medical equipment like blood pressure, and equipment for diabetes (glucometer). It will help us in the referrals. If at all we have such equipment and the community is aware- when someone has any of the signs, they can come to me and get the first test and I could refer them to the hospital.”

#### Barriers to a VHT role in preventing NCDs in communities

The major barriers participants reported were the lack of formal VHT education on NCDs, poor healthcare infrastructure, community poverty, discouraging attitudes from medical providers toward community members, and lack of assistance and support for VHTs from medical personnel (Table 5).

*“Lack of equipment like counseling cards also affects our work as VHTs. For instance, use of pictorial teaching materials in educating community members about NCDs will help to reinforce the knowledge/information the VHT is giving.”* FGD2, Participant 8

**Table 5:** Examplary statements on classification of barriers to VHT role in preventing NCDs in communities

Structural Barriers	Exemplary Statements
Lack of VHT education on NCDs	Ppt#1-FGD4 “There is a lack of knowledge on the causes, signs and symptoms of NCDs amongst us VHTs, so it becomes a barrier in dealing with NCDs as one will be dealing with something we don’t know of.”
Lack of medical services, medications, equipment, personnel	Ppt#5-FGD2 “Sometimes, we refer people to the hospitals but they return without medications which affect our VHT work and also the community’s dealings with VHT workers.”
Lack of assistance/supervision from medical personnel	Ppt#3- FGD4 “Medical personnel are not monitoring or supervising VHTs.”
Functional barriers	
Lack of transport to medical facilities	Ppt#5- FGD2 “Lack of equipment and means of transport (bicycle) affect our work, probably more in the rainy season.”
Community’s lack of money for medical services	Ppt#2- FGD1 “Due to lack of money, they (community) have no transport to reach the hospital and pay for the tests.”
Discouraging attitude of medical personnel towards community members	Ppt#11- FGD1 “Health workers scare off patients when the patients come in more than once if I as a VHT refer a patient to the hospital, the medical worker gives them medication. If the patient’s condition worsens, as a VHT, I encourage them to go back but the medical personnel complain that this particular patient would be depriving others from getting medication.”
Lack of monetary encouragement from organizations to aid VHT work	Ppt#2- FDG2 “Although we are not working for a salary, at least once in a while, we should be given something.”
Lack of VHT recognition by medical personnel	Ppt#11- FGD1 “It also hurts when I refer someone to the hospital with a reference letter and the medical officer throws out my reference letter.”
Community non-adherence to VHT recommendations	Ppt#7- FGD3 “We refer people to hospital and they don’t go. At one time, I gave transport to a client to go to the hospital but this person did not go. It hurts me as a VHT.”
Lack of VHT equipment/medication	Ppt#8- FGD3 “Lack of equipment such as BP machines, machines for testing diabetes. After having this equipment, people will come to me for help to get tested. With machines, they will trust the results and the information I give them; if I talk without proof or on assumption, they will be doubtful and ask for proof.”

VHT members identified the interconnectedness of these barriers. For instance, respondents stated that when they refer community members to the health centers for NCD-related issues, they encounter negative attitudes from medical personnel, or are not provided with testing or medications for their condition. Such members return to their communities reluctant to seek care either by VHTs or at health centers.

*“People go to the hospital and want to test for diseases but are not being rendered these services. There is poor management in health units which affects our work at the end of the day. When we refer other people, they refer to the failure of their (community) members to get the services they needed at the health units.”* FGD1, Participant 10

## Discussion

Our findings reveal that Ugandan VHT members possess some knowledge and awareness of NCDs and associated risk factors but identify a lack of NCD knowledge in their communities. Participants saw a potential role for themselves as conduits of knowledge to their communities about NCDs, on the condition that they were empowered with knowledge through training and support from medical personnel. VHT members were also able to articulate potential facilitators of, and barriers to, incorporating NCD care into their existing roles.

### VHT understanding of NCDs

Participants understood that NCDs were not transmissible and spoke of risk behaviors responsible for NCDs, although their knowledge of disease-specific risk factors and characteristics was less well developed. For example, while almost 70% of participants described knowing ‘a little’ about diabetes, less than half of those surveyed were aware that diabetes is preventable. We hypothesize that VHT members acquired their rudimentary knowledge through exposure to the healthcare system including occasional training workshops, community members with NCDs, and other NCD-related research activities occurring in IMHDSS. Many were confident that they were already encountering community members with NCDs while performing their VHT work. Given the local epidemiologic data on the NCDs they identified, such as hypertension, diabetes, and asthma, which are becoming widespread among their communities, [7–9] we expect that this perception was accurate. These community members are receiving diagnoses at health facilties and sporadic community-based screening campaigns. Indeed, self-reported knowledge of NCDs tended to be in line with prevalence estimates reported by the Ugandan STEPS survey [7, 9]; more VHT members reported knowledge of high blood pressure than diabetes, reflecting the higher prevalence of hypertension among their community. Future research should seek to gain a more comprehensive understanding of VHT practices regarding identifying persons with NCDs and referrals to health facilties.

While they themselves reported being aware of NCDs, VHT members linked the lack of NCD awareness among members of their communities to the latent clinical nature of NCDs and the lack of availability of screening services at community health centers. A systematic review of prior research on access to care for conditions including malaria, pneumonia, obstetric and gynecological disorders, malnutrition and HIV/AIDS in Uganda has shown that lack of awareness and knowledge about health conditions, along with perceived poor quality of health services and a perceived poor attitude of health workers, are demand-side barriers to utilization of health services [20]. Similar findings may be true for NCDs and warrant exploration. Such lack of awareness within communities, if present for NCDs, may inhibit the development of the peoples’ voice, of health advocates demanding systemic change to improve services. The VHT members in this study appeared interested in advocating for the improvement of NCD services on behalf of their communities, though expressed concerns that their voices would not be heard by facility-based health professionals.

### VHTs role in preventing/treating NCDs in communities

Several interventions have shown the benefits of using CHW-medical personnel partnerships to promote NCD prevention and management in communities. In American Samoa, an intervention that employed nurse-community health teams in diabetes care more than doubled the odds of reducing HbA1c levels by at least 0.5% among the intervention group compared with those receiving usual care [21]. A multi-LMIC study which involved medical partnership via training and supervision of CHWs, improved CHWs’ ability to assess cardiovascular risk in their communities. This in turn increased disease detection and diagnosis [22].

CHWs have also served in successful roles for NCD care and prevention as providers of direct services to clients, monitors of clients’ care, peer educators for newer CHWs, and as administrators, overseeing reporting and documenting of clients’ care [21–23]. For example, data from a study in Iran showed that having CHWs engage in diabetes prevention and control lowered participants’ fasting blood glucose [24]. These CHWs were able to conduct training sessions for high-risk individuals on adopting healthy lifestyles and diets. Medical personnel also visited local communities to screen and treat for diabetes. By working together, medical personnel and CHWs were able to keep high-risk individuals in care. In the present study, VHTs noted that they already engage in some of these activities, such as referral of clients, outside of their formal VHT roles. They also advocated for similar VHT-clincian collaborations to improve NCD detection and care.

Indeed, VHT members interviewed thought their present roles in helping community members access health knowledge, care, and services for maternal-child health and communicable diseases could be replicated for NCD prevention. They were enthusiastic about the expansion of their role into this area on the condition that they receive adequate training, and that screening services and proper referral and reporting systems were available to their communities. This finding is supported by a previous study in South Africa, where CHWs were involved in ongoing care of community members with NCDs. That study recommended context-relevant and organized education for VHTs and the provision of resources to build CHW capacity in NCDs as significant facilitators for CHWs delivering NCD services [23].

Frequently interwoven into the VHT members’ responses to facilitators of and barriers to VHT roles in NCD screening and care was the strong desire for medical personnel to collaborate and support VHTs to promote and validate their work in the communities. They believed it would be beneficial for medical personnel to intiate conversations about NCDs in their communities and create safe spaces for addressing NCDs by visibly being involved in preventive efforts within the communities. The Community Health System Strenghthening Model, used in other sub-Saharan African settings, has been successful at bringing community representatives, CHW, and health facilty staff together to form teams that address challenges such as these raised by the VHT. Future research might study this model in the context of enhancing community-facility engagement around NCD management [25].

### Barriers to VHT roles in preventing NCDs in communities

VHT members spoke to the cycle of neglect where several barriers such as lack of services and medical personnel would hinder preventive efforts at the community level. These were considered more critical barriers to the expanstion of the VHT role than the lack of financial incentivization for VHT work. A lack of financial support has been identified as a major factor limiting CHW motivation in prior research [26, 27] , though our findings suggest otherwise and are consistent with another recent study among CHWs in Uganda, which described community relationships and trust as being the most important motivators for CHW work [28]. Participants in the present study described how they would be motivated by greater availibility of equipment, uniforms, and transportation, while payment for their work was very rarely mentioned. In LMICs, primary care systems are faced with unavailability of basic diagnostic instruments and services for NCD screening and detection, poor access to medicines for treating NCDs, a shortage of healthcare professionals to manage NCDs, and poor reporting and referral systems [29], all of which VHTs believed needed to be addressed in order for them to fulfil a role in NCD prevention and treatment. They were perceptive in noting that, beyond their own access to equipment and diagnositic instruments, if the health facilities to which they were referring clients did not have the resources to provide effective and timely treatment, then their role would be undermined and the community would become mistrustful of their recommendations to seek addditonal care. Innovative financing models, such as public-private partnerships and taxation of alcohol, tobacco, or sugar-sweetened beverages, have been proposed to support the costs of NCD program expansion [30].

VHTs already play critical roles in the delivery of primary health services and have broad geographic coverage and opportunities for individual interactions with high-risk individuals within their communities [16]. Task-shifting or role expansion has inherent challenges such as overburdening health workers [31]. However, our study participants did not express concerns regarding expanding their roles to include NCDs. Given their expressed willingness and motivation to fulfil a role in NCD prevention and care, VHTs appear to represent an ideal human capacity to implement primary NCD interventions in Uganda [16]. Investment in further training and NCD education for VHTs and their role in delivering NCD prevention education and NCD care should be considered as national and regional NCD frameworks are developed [32].

### Limitations and strengths

While our study elucidated the potential role of VHTs in NCD prevention and treatment in Uganda, there were some limitations to our approach that should be acknowledged. Our KAP questionnaire, while successful in measuring general NCD knowledge and VHT attitudes related to NCD risk factors, was less robust for measuring disease-specific knowledge, knowledge of diet and physical activity-related risk factors for NCDs and current practices for prevention or treatment. Additionally, since no locally validated questionnaires were available for use in this setting, we relied on questionnaires validated for use in other settings. Generalizability may also be an issue; the VHT program in IMHDSS is currently better developed than in other regions, so the applicability of our findings to other regions in Uganda might be limited. Finally, VHT members in IMHDSS may have greater exposure to NCD-related issues than VHT members elsewhere in Uganda by virtue of other research projects such as “A people-centered approach through Self Management And Reciprocal learning for the prevention and management of Type 2 Diabetes” (SMART2D) that was in its early stages at the time of the current study.

A major strength of this study, however, was the use of a mixed methods approach to explore existing knowledge and perceptions about NCDs among the Ugandan VHTs. This allowed us to elicit complementary information via the questionnaire and FGDs about understanding of NCDs and perceptions of a potential VHT role in preventing them.

## Conclusions

In this study we have shown that Ugandan VHT members already possess some knowledge and understanding of NCDs, especially around the mode of transmission, diet-related risk factors, and the late manifestation of NCD symptoms, although gaps remain. VHT members acknowledged the prescence of NCDs in their communities and identified their potential role as connecting their communities to knowledge and screening services. They displayed a willingness and motivation to engage in preventive efforts directed at NCDs and identified VHT and community education on NCDs and a strong presence of medical personnel in their communities as being important facilitators of their ability to fulfil this role. Specifically, participants expressed a desire to develop strong alliances with facility-based healthcare providers; to feel legitiamized by, and integrated within, the health system at large. These results should be used to inform future research and policy that seeks to develop the role of VHTs as it relates to community-based NCD prevention efforts in Uganda.

## Abbreviations

CHWs: Community Health Workers
CVD: Cardiovascular Diseases
FGDs: Focus Group Discussions
IMHDSS: Iganga-Mayuge Health and Demographic Surveillance Site
KAP: Knowledge, Attitudes and Perception
LMICs: Low and Middle Income Countries
MOH: Ministry of Health
NCDs: Non-Communicable Diseases
SES: Socioeconomic status
SMART2D: Self Management Approach and Reciprocal Learning for Type 2 Diabetes
STEPS: STEPwise Approach to Surveillance
VHTs: Village Health Teams
WHO: World Health Organization
